# Sleep Phenotype, Shift-Related Occupation, and Coronary Plaque Complexity in Young Adults: Association with CT-Derived Functional Ischemia

**DOI:** 10.5334/gh.1586

**Published:** 2026-09-22

**Authors:** Yuhuan Chen, Qi Zhao, Jiangbo Gao, Ruoxi Zhang, Jian Yang

**Affiliations:** 1 Department of Radiology, Xi’an Jiaotong University, Xi’an 710061, China; 2 Department of Medicine, Keya Medical Technology CO. Ltd, Beijing 1001176, China; 3 Department of Cardiology, the First Affiliated Hospital of Harbin Medical University, Harbin 150001, China; 4 Department of Cardiology, Xi’an Changan Hospital, Xi’an 710016, China; 5 Department of Cardiology, Suzhou Kowloon Hospital, School of Medicine Shanghai Jiaotong University, Suzhou 215000, China; 6 Department of Radiology, the First Affiliated Hospital, Xi’an Jiaotong University, Xi’an 710061, China

**Keywords:** Sleep phenotype, Shift-related occupation, Coronary plaque complexity, CT-derived fractional flow reserve, Functional ischemia, Young adults

## Abstract

**Background:**

Sleep disruption and irregular work schedules are increasingly recognized as behavioral risk factors for premature cardiovascular disease. Their associations with coronary plaque complexity and CT-derived functional ischemia in young adults remain underexplored.

**Objective:**

To investigate the association of multidimensional sleep phenotype, shift-related occupation, and coronary plaque complexity as well as CT-derived functional ischemia in adults aged ≤40 years, adjusting for clinical confounders.

**Methods:**

This retrospective study included 3,983 young adults who underwent coronary CT angiography (CCTA). Sleep phenotype was assessed using a multidimensional score incorporating sleep duration, timing, regularity, quality, and sleep-related symptoms. Occupations were classified as shift-related or non-shift. Coronary plaque characteristics, including stenosis severity, minimum lumen area, lesion length, diffuse disease, occlusion, coronary artery calcium score, and CT-derived fractional flow reserve (CT-FFR), were analyzed. Multivariable logistic regression, adjusted for age, sex, BMI, smoking, hypertension, diabetes, dyslipidemia, and shift-related occupation, assessed associations with CT-FFR ≤ 0.80.

**Results:**

Poor sleep phenotype was associated with more severe stenosis, smaller lumen area, longer lesions, and higher prevalences of diffuse disease, occlusion, and CT-FFR ≤ 0.80. In multivariable analysis, sleep phenotype remained independently associated with CT-FFR ≤ 0.80 after adjusting for clinical covariates and shift-related occupation. Shift-related occupation further modified this association, with a stronger relationship observed among shift workers.

**Conclusions:**

In young adults, poorer sleep phenotype was accompanied by more complex coronary plaque features and was independently associated with a higher likelihood of CT-FFR-defined functional ischemia after adjustment for clinical covariates.

**Lay Summary:**

Poor sleep and irregular working hours may affect heart health, but their relationship with early coronary artery disease in young adults is not well understood. We studied 3,983 adults aged 40 years or younger who underwent coronary CT scans. Participants with poorer sleep patterns were more likely to have longer and more severe coronary artery narrowing, more widespread plaque, and reduced blood flow to the heart. These associations remained after accounting for established cardiovascular risk factors. The relationship between poor sleep and impaired coronary blood flow was particularly strong among people working night shifts or other irregular schedules. Although this observational study cannot prove that poor sleep directly causes coronary disease, the findings suggest that sleep habits and work schedules may help identify young adults who could benefit from earlier cardiovascular risk assessment and preventive support.

## Graphical Abstract

**Figure:**
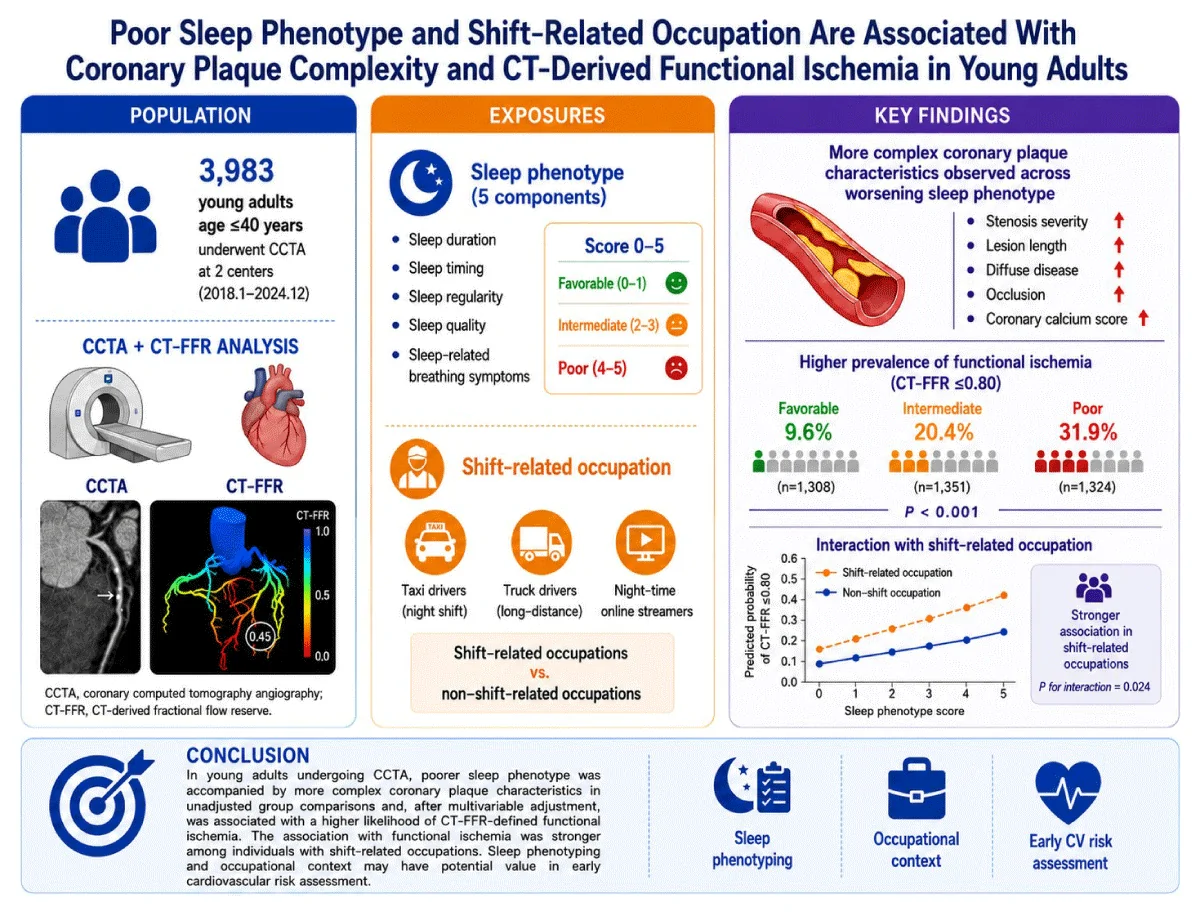


## Introduction

Premature coronary atherosclerosis is increasingly recognized as an important clinical issue in young adults (1). While traditional cardiovascular risk factors such as smoking, hypertension, diabetes, dyslipidemia, and obesity contribute to early coronary plaque formation, they only partially explain the onset of functional ischemia in this population (2). Identifying additional modifiable behavioral and environmental factors may improve early risk assessment and preventive strategies (3,4).

Sleep disturbance has emerged as a potential behavioral factor associated with cardiovascular risk (5). Features such as short or long sleep duration, irregular sleep timing, poor sleep quality, and sleep-disordered breathing may influence autonomic regulation, systemic inflammation, metabolic balance, endothelial function, and circadian alignment (6,7). These pathways could contribute to early coronary plaque development and adverse plaque phenotypes (8). However, the relationship between multidimensional sleep patterns and coronary plaque complexity in young adults has not been fully characterized (9,10).

Shift-related occupations represent another source of circadian disruption (11). Work schedules that include night shifts, long-distance driving, online content creation, and other irregular patterns may be associated with sleep loss, circadian misalignment, sedentary behavior, and psychosocial stress (12). These exposures may be particularly relevant in young adults, in whom lifestyle and occupational factors could precede clinically overt cardiovascular disease and influence early coronary phenotypes. Whether shift-related occupation modifies the association between sleep disturbance and CT-derived functional ischemia remains unclear.

Coronary computed tomography angiography (CCTA) provides a noninvasive approach to evaluate coronary plaque burden, stenosis severity, lesion length, diffuse disease, occlusion, and coronary artery calcium (13). CT-derived fractional flow reserve (CT-FFR) enables functional assessment of lesion-specific ischemia beyond anatomical stenosis (14). Integrating anatomical plaque features with CT-FFR allows for a more comprehensive assessment of early coronary disease.

In this study, we examined the associations of sleep phenotype and shift-related occupation with coronary plaque complexity and CT-derived functional ischemia in adults aged ≤40 years undergoing CCTA. We further explored the incremental value of sleep phenotype in predicting functional ischemia beyond traditional clinical risk factors and assessed whether shift-related occupation modifies these associations.

## Methods

### Study population

This retrospective observational study included consecutive patients aged ≤40 years who underwent CCTA for suspected coronary artery disease. The study protocol adhered to the ethical principles outlined in the 1975 Declaration of Helsinki and its later amendments. Approval was obtained from the Research Ethics Committee of Suzhou Kowloon Hospital, Shanghai Jiao Tong University School of Medicine (HG-2025-030), and the requirement for informed consent was waived due to the retrospective nature of the study. All data were retrieved from the imaging systems of each participating hospital for patients who had previously undergone CCTA scans.

**Figure 1:**
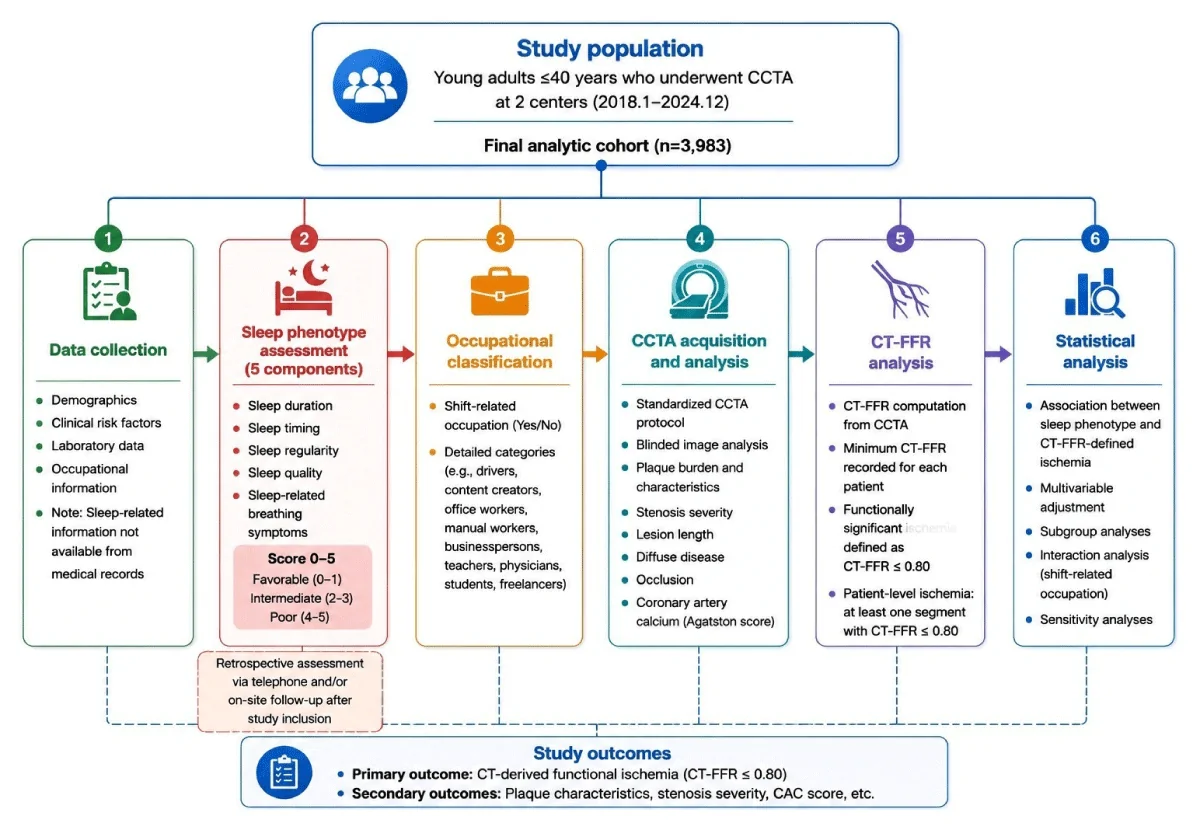
Study flowchart. CCTA, coronary computed tomography angiography; CT-FFR, computed tomography-derived fractional flow reserve.

Patients with prior coronary revascularization, known structural heart disease, or inadequate image quality were excluded. The final study population consisted of 3,983 patients (Figure 1). Clinical information, including demographics, cardiovascular risk factors, laboratory measurements, and occupational details, was collected from electronic medical records. Data extraction and handling were performed in a manner ensuring de-identification to maintain patient confidentiality. This dataset provided the basis for evaluating the association between multidimensional sleep phenotype, shift-related occupation, and coronary plaque complexity as well as CT-derived functional ischemia.

### Sleep phenotype assessment and data collection

After identifying the 3,983 eligible patients based on inclusion and exclusion criteria, sleep-related information was collected retrospectively through structured telephone interviews and in-person follow-up. Sleep phenotype was quantified using a multidimensional scoring system incorporating sleep duration, timing, regularity, quality, and sleep-related breathing symptoms (15,16). The sleep assessment was intended to characterize participants’ reported habitual sleep patterns. Information regarding the duration of individual sleep characteristics, cumulative years of exposure to a specific sleep phenotype, and longitudinal changes in sleep patterns was not systematically available; therefore, cumulative sleep exposure was not quantified in the present study.

Each component was assigned 1 point if the unfavorable condition was present and 0 points otherwise, with equal weighting across all five components. The total sleep phenotype score ranged from 0 to 5, with higher scores indicating poorer sleep patterns. This prespecified equal-weighting approach was not derived from CT-FFR outcomes in the present cohort and was adopted to maintain simplicity and interpretability, consistent with previously published multidimensional healthy sleep scoring frameworks. The five components were defined as follows: sleep duration: average sleep duration of <7 hours or >9 hours per night (17); late sleep timing (habitual late bedtime): usual sleep onset time later than 23:00 or frequent late sleeping (≥3 times per week) (16,18); irregular sleep pattern: variability in sleep timing (difference between weekdays and weekends ≥1 hour) or self-reported irregular sleep habits (18,19); poor sleep quality/insomnia symptoms: self-reported difficulty initiating or maintaining sleep, early awakening, or documented insomnia (16,18); and snoring or daytime sleepiness: presence of habitual snoring, suspected sleep-disordered breathing, or excessive daytime sleepiness (16,20). Sleep data were available for all 3,983 participants.

Based on the total score, patients were categorized into three groups: favorable sleep phenotype: 0–1 points; intermediate sleep phenotype: 2–3 points; and poor sleep phenotype: 4–5 points.

### Occupational classification

Occupational information was obtained from electronic medical records and patient-reported data at the time of CCTA examination. Occupations were categorized based on work schedule characteristics, with a focus on circadian rhythm disruption. Predefined occupational categories were established prior to analysis.

Shift-related occupations were defined as those involving irregular working hours, night-shift work, or frequent circadian misalignment. These included night-shift drivers (e.g., taxi and truck drivers) and night-time online streamers who frequently work during late hours. Non–shift-related occupations were defined as those with relatively regular daytime working schedules.

For descriptive analyses, occupational categories were further grouped into shift-related occupations (night-shift taxi drivers, night-shift truck drivers, and night-time online streamers) and non-shift-related occupations (college students, manual workers, office workers, businesspersons, teachers, physicians, and freelancers). This grouping clarifies which occupations were considered shift-related and provides the basis for subsequent analysis across sleep phenotype groups. For the primary analyses, occupational exposure was operationalized as a binary variable (shift-related vs non-shift-related) according to the predominant work schedule characteristics described above. Detailed quantitative measures of shift-work exposure, including night-shift frequency, hours worked per shift, duration of shift-work employment, and cumulative years of exposure to irregular work schedules, were not systematically available.

### CCTA acquisition and analysis

**Figure 2:**
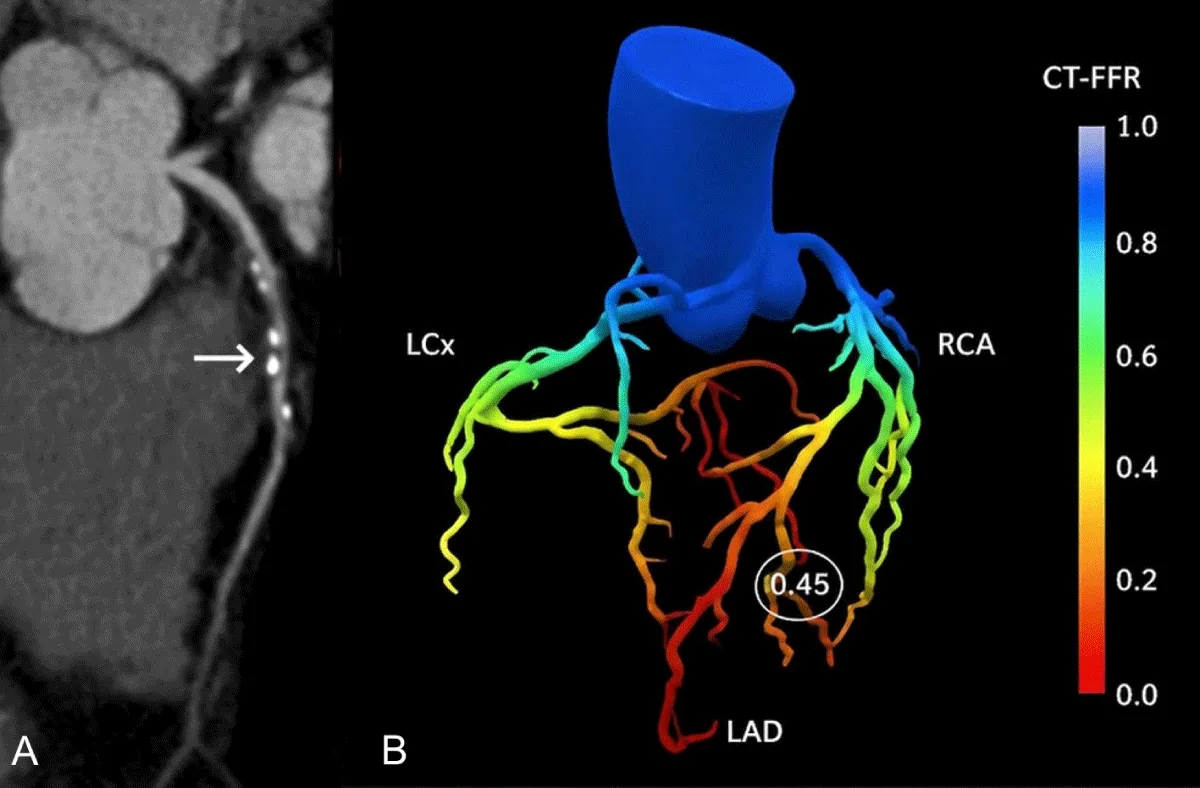
Representative case of CT-derived fractional flow reserve (CT-FFR) analysis. **(A)** Curved multiplanar reconstruction of coronary CT angiography shows a mixed plaque causing intermediate stenosis in the mid-left anterior descending artery (LAD) (arrow). **(B)** Three-dimensional CT-FFR model demonstrates a hemodynamically significant lesion in the mid-LAD with a CT-FFR value of 0.45 (circled), while other coronary segments show higher CT-FFR values.

CCTA was performed using a multi-detector CT scanner according to standard clinical protocols (21). Scanning parameters, including tube voltage, tube current, and contrast administration, were adjusted based on patient characteristics. Sublingual nitroglycerin was not administered before CCTA as part of the institutional scanning protocol. Electrocardiogram-gated image acquisition was applied, and images were reconstructed at optimal cardiac phases for coronary evaluation (Figure 2A). All CCTA datasets were analyzed using dedicated cardiovascular imaging software by experienced readers who were blinded to sleep phenotype and occupational information. Coronary artery segments were evaluated according to standard anatomical definitions.

Quantitative and qualitative plaque characteristics were assessed, including stenosis severity, minimum luminal area (MLA), lesion length, presence of diffuse disease, and occlusion. MLA was measured at the site of maximal stenosis for each coronary segment, and the minimum value across all assessed vessels was recorded for each patient. Diffuse disease was defined as involvement of a coronary segment ≥20 mm in length with continuous plaque presence without any focal stenosis ≥70%, providing a standardized threshold for long-segment disease. Coronary artery calcium was quantified using the Agatston scoring method. To ensure consistency, all measurements were performed following standardized analysis protocols. Discrepancies between readers were resolved by consensus discussion.

### Blinding and plaque assessment

All CCTA scans were independently read by two experienced cardiovascular imaging specialists (researchers trained in CCTA interpretation) who had no access to patients’ medical records, sleep phenotype, or occupational information. Plaque characteristics, including stenosis severity, minimum luminal area, lesion length, diffuse disease, and occlusion, were manually extracted by these readers using dedicated cardiovascular imaging software. Consensus readings were performed in cases of disagreement. To assess measurement reproducibility, a randomly selected subset of 100 CCTA examinations was independently reassessed by both cardiovascular imaging readers. Interobserver agreement for continuous variables, including stenosis severity, minimum lumen area, lesion length, and coronary artery calcium score, was evaluated using intraclass correlation coefficients (ICCs). Agreement for categorical variables, including diffuse disease and occlusion, was assessed using Cohen’s κ coefficients. Both readers remained blinded to clinical characteristics, sleep phenotype, and occupational information during the agreement assessment.

### CT-FFR analysis

CT-FFR was calculated from CCTA datasets using the DEEPVESSEL computational software (Keya Medical Technology Co., Ltd.), which includes a coronary artery segmentation model and a computational fluid dynamics simulation model validated in prior studies (13). Coronary artery lumen segmentation and centerline extraction were performed automatically, followed by computational assessment of pressure gradients along the coronary tree for all major epicardial vessels with diameters ≥1.8 mm. CT-FFR values were obtained for each analyzable coronary segment, and lesion-specific measurements were taken 20 mm distal to the site of maximal stenosis for each lesion. For patient-level analyses, the minimum CT-FFR value across all coronary segments was recorded to represent overall functional ischemic burden.

Functionally significant ischemia was defined as CT-FFR ≤ 0.80, consistent with established thresholds for lesion-specific ischemia. Patients with at least one segment meeting this criterion were classified as having CT-derived functional ischemia (14). All CT-FFR analyses were performed blinded to sleep phenotype and occupational classification (Figure 2B).

### Statistical analysis

Continuous variables are presented as mean ± standard deviation or median with interquartile range, as appropriate. Categorical variables are presented as counts and percentages. Differences among favorable, intermediate, and poor sleep phenotype groups were assessed using one-way ANOVA or Kruskal–Wallis tests for continuous variables and chi-square tests for categorical variables. Multivariable logistic regression was applied to evaluate the association between sleep phenotype and CT-derived functional ischemia (CT-FFR ≤ 0.80). The fully adjusted model simultaneously included sleep phenotype score, shift-related occupation, age, sex, body mass index, smoking status, hypertension, diabetes, and dyslipidemia. Smoking status, hypertension, diabetes, and dyslipidemia were entered as binary covariates based on the clinical information available at baseline. Quantitative measures of cumulative exposure or long-term disease control were not consistently available and were therefore not incorporated into the primary models.

As an exploratory analysis, the five individual components of the sleep phenotype were further evaluated to determine whether their associations with CT-derived functional ischemia differed. Each component—abnormal sleep duration, late sleep timing, irregular sleep pattern, poor sleep quality/insomnia symptoms, and snoring/daytime sleepiness—was first examined individually using logistic regression for CT-FFR ≤ 0.80. Multivariable models were subsequently adjusted for age, sex, BMI, smoking, hypertension, diabetes, dyslipidemia, and shift-related occupation. In addition, all five sleep components were entered simultaneously into a multivariable model to assess their mutually adjusted associations with CT-FFR ≤ 0.80. These component-level analyses were considered exploratory.

To evaluate the incremental predictive value of sleep phenotype, receiver operating characteristic (ROC) curve analysis was conducted. Model discrimination was compared among the clinical model, clinical model plus sleep phenotype, clinical model plus CCTA variables, and the full model including all covariates. Area under the curve (AUC) values and changes in AUC were reported. To examine whether occupational circadian disruption modified the relationship between sleep phenotype and CT-derived functional ischemia, an interaction term between sleep phenotype score and shift-related occupation was included in a multivariable logistic regression model. The model included sleep phenotype score, shift-related occupation, their interaction term, age, sex, BMI, smoking, hypertension, diabetes, and dyslipidemia. Stratified analyses were subsequently performed according to shift-related occupation status. All statistical tests were two-sided, with *P* < 0.05 considered statistically significant. Analyses were performed using SPSS version 26.0 (IBM Corp., Armonk, NY, USA) and R version 4.3.1 (R Foundation for Statistical Computing, Vienna, Austria).

## Results

### Baseline characteristics

A total of 3,983 young adults were included in the analysis and categorized into favorable sleep (n = 1,308), intermediate sleep (n = 1,351), and poor sleep phenotype groups (n = 1,324). Baseline demographic and clinical characteristics were generally comparable across the three groups. There were no significant differences in age, body mass index, smoking status, hypertension, diabetes, dyslipidemia, lipid profiles, HbA1c, hs-CRP, or uric acid levels among the groups. However, the proportion of male patients increased across favorable, intermediate, and poor sleep phenotype groups (64.6%, 68.7%, and 72.0%, respectively; *P* < 0.001) (Table 1).

**Table 1:** Baseline characteristics stratified by sleep phenotype.


VARIABLE	FAVORABLE SLEEP (n = 1308)	INTERMEDIATE SLEEP (n = 1351)	POOR SLEEP (n = 1324)	*P*-VALUE

Age (years)	29.94 ± 6.06	29.93 ± 6.07	30.07 ± 6.14	0.795

Male sex, n (%)	845 (64.6%)	928 (68.7%)	953 (72.0%)	<0.001

BMI (kg/m²)	25.87 ± 3.22	25.96 ± 3.14	25.96 ± 3.15	0.705

Smoking, n (%)	795 (60.8%)	785 (58.1%)	797 (60.2%)	0.333

Hypertension, n (%)	866 (66.2%)	873 (64.6%)	887 (67.0%)	0.418

Diabetes, n (%)	18 (1.4%)	11 (0.8%)	15 (1.1%)	0.220

Dyslipidemia, n (%)	869 (66.4%)	852 (63.1%)	865 (65.3%)	0.177

Total cholesterol (mmol/L)	4.52 ± 0.80	4.52 ± 0.82	4.48 ± 0.80	0.344

LDL-C (mmol/L)	3.02 ± 1.13	3.02 ± 1.08	3.01 ± 1.09	0.907

HDL-C (mmol/L)	1.31 ± 0.50	1.29 ± 0.50	1.30 ± 0.50	0.801

Triglyceride (mmol/L)	2.25 ± 0.93	2.24 ± 0.93	2.23 ± 0.92	0.818

HbA1c (%)	6.52 ± 1.60	6.49 ± 1.58	6.47 ± 1.62	0.676

hs-CRP (mg/L)	11.54 ± 2.76	11.53 ± 2.85	11.40 ± 2.79	0.346

Uric acid (μmol/L)	458.33 ± 31.26	458.06 ± 30.89	457.42 ± 30.84	0.744


Mean values ± standard deviation, median (interquartile range), and n (%) were reported for variables, respectively. BMI, body mass index; HDL, high density lipoprotein; HbA1c, glycosylated hemoglobin; hs-CRP, high-sensitivity C-reactive protein; LDL, low-density lipoprotein.

### CCTA findings across sleep phenotype groups

Coronary plaque characteristics differed significantly across sleep phenotype groups (Table 2). Patients with a poorer sleep phenotype had greater stenosis severity, smaller minimum lumen area, and longer lesion length compared with those with more favorable sleep patterns (all *P* < 0.001). The prevalence of diffuse coronary lesions increased from 9.0% in the favorable sleep group to 21.9% in the intermediate sleep group and 41.3% in the poor sleep group, while occlusion increased from 1.7% to 4.5% and 10.0%, respectively (both *P* < 0.001).

**Table 2:** Coronary CTA characteristics according to sleep phenotype.


VARIABLE	FAVORABLE SLEEP (n = 1308)	INTERMEDIATE SLEEP (n = 1351)	POOR SLEEP (n = 1324)	*P*-VALUE

Degree of stenosis (%)	34.2 ± 18.6	42.8 ± 21.4	51.6 ± 24.3	<0.001

Minimum lumen area (mm²)	10.8 ± 4.2	8.6 ± 3.7	6.9 ± 3.1	<0.001

Lesion length (mm)	11.2 ± 6.1	15.6 ± 8.4	21.3 ± 10.7	<0.001

Diffuse lesion, n (%)	118 (9.0%)	296 (21.9%)	547 (41.3%)	<0.001

Occlusion, n (%)	22 (1.7%)	61 (4.5%)	132 (10.0%)	<0.001

Calcium volume (mm^3^)	18.4 (0–42.6)	21.6 (0–58.3)	24.9 (0–71.4)	0.071

CACS (Agatston)	5.2 (0–38.1)	8.9 (0–64.2)	14.6 (0–102.5)	<0.001

Mean calcium attenuation (HU)	612 ± 164	655 ± 181	701 ± 196	<0.001

CT-FFR ≤ 0.80, n (%)	126 (9.6%)	276 (20.4%)	422 (31.9%)	<0.001


Mean values ± standard deviation, median (interquartile range), and n (%) were reported for variables, respectively. CTA, coronary computed tomography angiography; CACS, coronary artery calcification score; FFR, fractional flow reserve.

With respect to coronary calcification, calcium volume showed a nonsignificant increasing trend across groups, whereas CACS and mean calcium attenuation increased significantly with worsening sleep phenotype (both *P* < 0.001). The prevalence of CT-derived functional ischemia, defined as CT-FFR ≤ 0.80, also increased stepwise across favorable, intermediate, and poor sleep groups: 9.6%, 20.4%, and 31.9%, respectively (*P* < 0.001). These findings indicate a stepwise worsening of coronary plaque complexity and functional ischemia with increasing sleep disturbance.

### Association between sleep phenotype and CT-FFR ≤ 0.80

The prevalence of CT-derived functional ischemia (CT-FFR ≤ 0.80) increased stepwise across sleep phenotype groups, from 9.6% in the favorable group to 20.4% in the intermediate group and 31.9% in the poor sleep group (*P* < 0.001) (Table 2). In multivariable logistic regression analysis, sleep phenotype remained independently associated with CT-derived functional ischemia after adjustment for age, sex, BMI, smoking, hypertension, diabetes, dyslipidemia, and shift-related occupation. Each 1-point increase in sleep phenotype score was associated with a higher likelihood of CT-FFR ≤ 0.80 (adjusted OR 1.38, 95% CI 1.12–1.71, *P* = 0.003). Shift-related occupation was also independently associated with CT-FFR ≤ 0.80 (adjusted OR 1.67, 95% CI 1.21–2.30, *P* = 0.002) (Table 3).

**Table 3:** Multivariable logistic regression analysis outcome: CT-FFR ≤ 0.80.


VARIABLE	ADJUSTED OR (95% CI)	*P*-VALUE

Sleep phenotype score, per 1-point increase	1.38 (1.12–1.71)	0.003

Shift-related occupation	1.67 (1.21–2.30)	0.002

Age, per year	1.01 (0.99–1.02)	0.284

Male sex	1.12 (0.85–1.48)	0.421

Smoking	1.19 (0.91–1.56)	0.204

BMI, per kg/m²	1.03 (0.99–1.07)	0.118

Hypertension	1.09 (0.82–1.45)	0.561

Diabetes	1.36 (1.02–1.82)	0.037

Dyslipidemia	1.08 (0.81–1.44)	0.602


CT-FFR, computed tomography-derived fractional flow reserve; OR, odds ratio; CI, confidence interval; BMI, body mass index. The multivariable model was adjusted simultaneously for age, sex, BMI, smoking, hypertension, diabetes, dyslipidemia, sleep phenotype score, and shift-related occupation.

### Interobserver agreement for CCTA measurements

Interobserver agreement was assessed in a randomly selected subset of 100 patients. Agreement was excellent for stenosis severity (ICC, 0.931), minimum lumen area (ICC, 0.918), lesion length (ICC, 0.894), and coronary artery calcium score (ICC, 0.956). Agreement for diffuse disease and occlusion was also high, with Cohen’s κ coefficients of 0.872 and 0.846, respectively (Supplementary Table 1).

### Association of individual sleep components with CT-derived functional ischemia

Exploratory component-level analyses demonstrated heterogeneity in the magnitude of associations between individual sleep characteristics and CT-FFR ≤ 0.80. When evaluated individually and after adjustment for age, sex, BMI, smoking, hypertension, diabetes, dyslipidemia, and shift-related occupation, all five sleep components showed significant associations with CT-derived functional ischemia.

When all five sleep components were entered simultaneously into the multivariable model, irregular sleep pattern showed the strongest association with CT-FFR ≤ 0.80 (adjusted OR, 1.41; 95% CI, 1.18–1.68; *P* < 0.001), followed by late sleep timing (adjusted OR, 1.29; 95% CI, 1.08–1.54; *P* = 0.005) and abnormal sleep duration (adjusted OR, 1.24; 95% CI, 1.04–1.48; *P* = 0.016). In contrast, the associations of poor sleep quality/insomnia symptoms (adjusted OR, 1.19; 95% CI, 0.99–1.43; *P* = 0.061) and snoring/daytime sleepiness (adjusted OR, 1.14; 95% CI, 0.95–1.37; *P* = 0.153) were attenuated and were no longer statistically significant after mutual adjustment. Detailed results are presented in Supplementary Table 2.

### Incremental value analysis

The addition of sleep phenotype improved the predictive performance for CT-derived functional ischemia. Compared with the clinical model alone, which showed limited discrimination, adding sleep phenotype increased the AUC from 0.562 to 0.675, resulting in a significant improvement in discrimination. This improvement was also supported by significant net reclassification improvement and integrated discrimination improvement.

The model incorporating clinical and CCTA variables achieved a higher AUC of 0.738. The full model, which integrated clinical variables, sleep phenotype, shift-related occupation, and CCTA features, showed the best overall performance, with an AUC of 0.748 and significant improvements in ΔAUC, NRI, and IDI compared with the clinical model (Table 4). These findings suggest that sleep phenotype provides incremental information beyond traditional clinical risk factors. However, the increase in discrimination from the clinical plus CCTA model (AUC 0.738) to the full model (AUC 0.748) was modest, indicating that much of the predictive information for CT-FFR ≤ 0.80 was already captured by structural CCTA characteristics.

**Table 4:** Incremental value analysis.


MODEL	AUC (95% CI)	ΔAUC	*P*-VALUE	NRI (95% CI)	*P*-VALUE	IDI (95% CI)	*P*-VALUE

Clinical model	0.562 (0.544–0.580)	Reference	—	Reference	—	Reference	—

Clinical + sleep phenotype	0.675 (0.658–0.692)	0.113	<0.001	0.212 (0.156–0.268)	<0.001	0.046 (0.031–0.061)	<0.001

Clinical + CCTA variables	0.738 (0.722–0.754)	0.176	<0.001	0.348 (0.291–0.405)	<0.001	0.083 (0.064–0.102)	<0.001

Full model	0.748 (0.732–0.764)	0.185	<0.001	0.371 (0.312–0.430)	<0.001	0.091 (0.071–0.111)	<0.001


ΔAUC, NRI, and IDI were calculated relative to the clinical model. The clinical model included age, sex, BMI, smoking, hypertension, diabetes, and dyslipidemia. CCTA variables included stenosis severity, minimum lumen area, lesion length, diffuse lesion, occlusion, and CACS. AUC, area under the receiver operating characteristic curve; NRI, net reclassification improvement; IDI, integrated discrimination improvement; CCTA, coronary computed tomography angiography; BMI, body mass index; CACS, coronary artery calcium score.

### Occupational distribution across sleep phenotype groups

**Figure 3:**
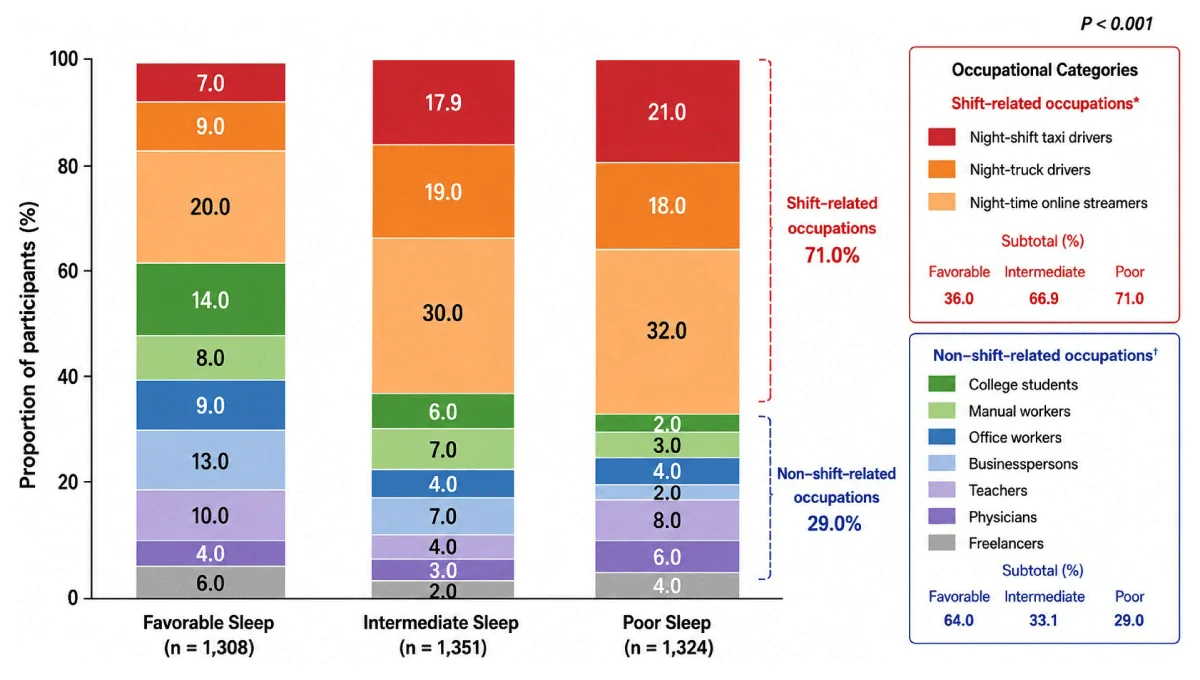
Distribution of occupational categories across sleep phenotype groups. *Shift-related occupations include night-shift taxi drivers, night-truck drivers, and night-time online streamers. †Non-shift-related occupations include college students, manual workers, office workers, businesspersons, teachers, physicians, and freelancers. Percentages may not sum to 100% due to rounding.

Occupational distribution differed significantly across sleep phenotype groups (*P* < 0.001). Shift-related occupations were more common among patients with intermediate and poor sleep phenotypes, particularly night-shift taxi drivers, truck drivers, and online content creators. In contrast, occupations with relatively regular schedules, such as college students, office workers, and businesspersons, were more frequent in the favorable sleep group (Table 5 and Figure 3). These findings indicate that occupational circadian disruption may represent an important contextual factor associated with poor sleep phenotype in young adults.

**Table 5:** Distribution of occupational categories across sleep phenotype groups.


VARIABLE	FAVORABLE SLEEP (n = 1308)	INTERMEDIATE SLEEP (n = 1351)	POOR SLEEP (n = 1324)	*P*-VALUE

Shift-related occupations				

Night-shift taxi drivers	92 (7.0%)	242 (17.9%)	278 (21.0%)	

Night-truck drivers	118 (9.0%)	257 (19.0%)	238 (18.0%)	

Night-time online streamers	261 (20.0%)	405 (30.0%)	424 (32.0%)	

Non-shift-related occupations				

College students	183 (14.0%)	81 (6.0%)	27 (2.0%)	

Manual workers	105 (8.0%)	95 (7.0%)	40 (3.0%)	

Office workers	118 (9.0%)	54 (4.0%)	53 (4.0%)	

Businesspersons	170 (13.0%)	95 (7.0%)	26 (2.0%)	

Teachers	131 (10.0%)	54 (4.0%)	106 (8.0%)	

Physicians	52 (4.0%)	41 (3.0%)	79 (6.0%)	

Freelancers	78 (6.0%)	27 (2.0%)	53 (4.0%)	

Overall comparison	—	—	—	<0.001


Categories were grouped for descriptive purposes based on predominant work schedule patterns.

### Predicted probability of CT-FFR ≤ 0.80 according to sleep phenotype and occupational status

**Figure 4:**
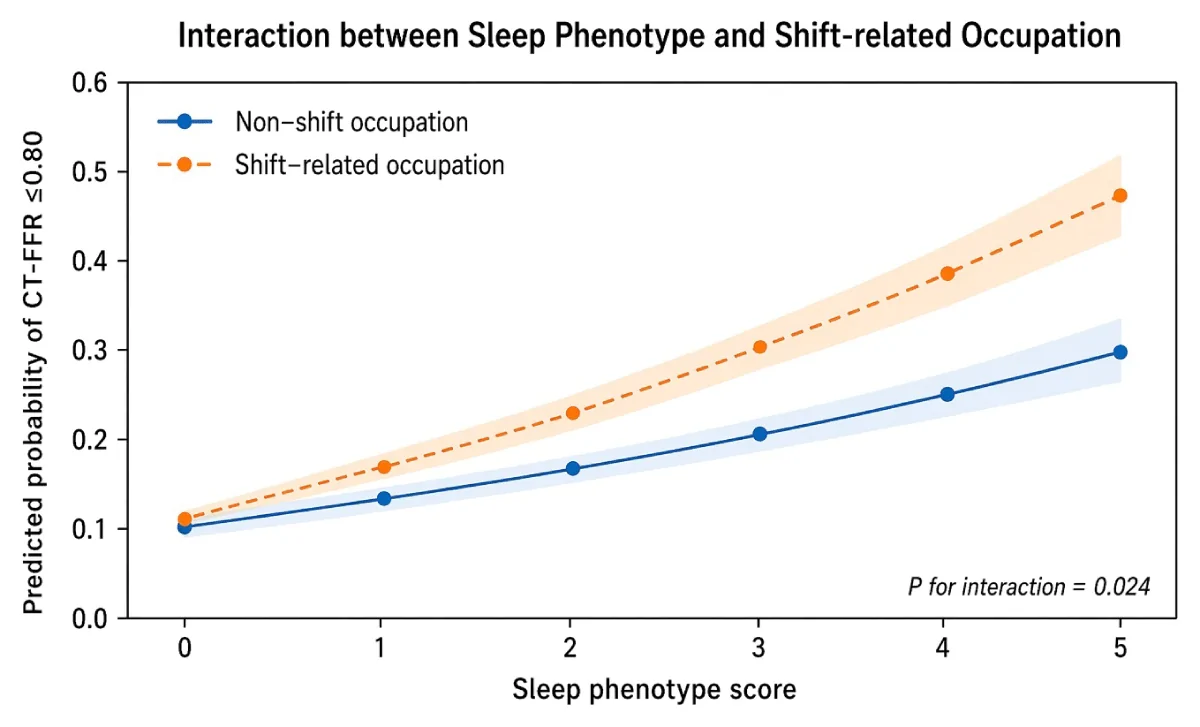
Predicted probability of CT-derived fractional flow reserve (CT-FFR) ≤ 0.80 according to sleep phenotype score stratified by occupational status. Predicted probabilities were derived from the multivariable interaction model including sleep phenotype score, shift-related occupation, the sleep phenotype × shift-related occupation interaction term, age, sex, body mass index, smoking, hypertension, diabetes, and dyslipidemia. Shaded areas represent 95% confidence intervals. CCTA, coronary computed tomography angiography; CT-FFR, computed tomography-derived fractional flow reserve.

In the interaction analysis, both sleep phenotype and shift-related occupation were associated with CT-derived functional ischemia. Importantly, the interaction term between sleep phenotype and shift-related occupation was statistically significant, suggesting that shift-related occupation modified the association between poor sleep phenotype and CT-FFR ≤ 0.80. In stratified analyses, the association between sleep phenotype and CT-FFR ≤ 0.80 appeared stronger among patients with shift-related occupations than among those with non–shift-related occupations (Table 6). These findings were further supported by model-based probability estimates, demonstrating a steeper increase in ischemia risk with worsening sleep phenotype among shift-related occupations (Figure 4).

**Table 6:** Interaction analysis between sleep phenotype and shift-related occupation.


VARIABLE	OR (95% CI)	*P*-VALUE

Sleep phenotype (per 1-point increase)	1.31 (1.08–1.59)	0.006

Shift-related occupation	1.54 (1.12–2.13)	0.008

Sleep × Shift occupation (interaction)	1.18 (1.02–1.37)	0.024

**Stratified Analysis**		

Non-shift occupation	1.25 (0.99–1.54)	0.081

Shift-related occupation	1.47 (1.18–1.83)	<0.001


OR, odds ratio; CI, confidence interval; BMI, body mass index. The interaction model included sleep phenotype score, shift-related occupation, the sleep phenotype × shift-related occupation interaction term, age, sex, BMI, smoking, hypertension, diabetes, and dyslipidemia. Stratified analyses were adjusted for age, sex, BMI, smoking, hypertension, diabetes, and dyslipidemia.

## Discussion

In this study of 3,983 young adults undergoing coronary computed tomography angiography, we found that poor sleep phenotype was associated with greater coronary plaque complexity and a higher prevalence of CT-derived functional ischemia. The proportion of patients with CT-FFR ≤ 0.80 increased stepwise across favorable, intermediate, and poor sleep phenotype groups, and sleep phenotype remained independently associated with functional ischemia after multivariable adjustment for potential confounders, including age, sex, BMI, smoking, hypertension, diabetes, dyslipidemia, and shift-related occupations. However, this association should not be interpreted as demonstrating a functional effect independent of structural coronary disease. The observed differences in plaque characteristics across sleep phenotype groups were based on unadjusted comparisons and should therefore be interpreted descriptively.

The primary sleep phenotype score used an equal-weighting approach, with each unfavorable sleep characteristic contributing one point. This approach was prespecified and was not derived from associations with CT-FFR outcomes in the present cohort, thereby maintaining the simplicity and interpretability of the composite score while reducing the potential for outcome-driven overfitting. Nevertheless, exploratory component-level analyses suggested heterogeneity in the magnitude of associations across individual sleep dimensions. Irregular sleep pattern showed the strongest association with CT-derived functional ischemia after mutual adjustment, followed by late sleep timing and abnormal sleep duration, whereas the associations of poor sleep quality/insomnia symptoms and snoring/daytime sleepiness were attenuated after simultaneous adjustment for the other sleep components. These findings should be considered hypothesis-generating rather than evidence for reweighting the current sleep phenotype score. Future studies with independent validation cohorts are warranted to determine whether empirically derived or externally validated weighted sleep scores provide additional value beyond a simple equally weighted composite measure.

Shift-related occupation modified this relationship, with a stronger association observed among individuals exposed to occupational circadian disruption. Our findings extend prior evidence linking sleep disturbance to cardiovascular risk (22) by demonstrating progressively adverse coronary plaque characteristics across sleep phenotype groups and an independent association between poorer sleep phenotype and CT-derived functional ischemia. The adjusted analysis applies specifically to CT-FFR-defined functional ischemia.

Several biological mechanisms may underlie these observations. Poor sleep duration, irregular sleep timing, and impaired sleep quality have been associated with sympathetic overactivity, systemic inflammation, insulin resistance, endothelial dysfunction, and impaired vascular repair processes (23,24). Circadian misalignment may further exacerbate metabolic and hemodynamic stress (25). These pathways may promote plaque progression, diffuse atherosclerosis, and adverse coronary remodeling, which may in turn contribute to lower CT-FFR values through their effects on epicardial coronary anatomy and lesion geometry. The relatively stronger associations observed for irregular sleep pattern and late sleep timing in the exploratory component-level analysis are consistent with a potentially important role of circadian disruption; however, mechanistic inference cannot be established from the present observational data. The present findings therefore do not establish a direct functional effect of sleep disturbance independent of structural coronary disease. Structural plaque characteristics may account for an important proportion of the observed association between sleep phenotype and CT-FFR, consistent with the relatively modest incremental discrimination of the full model beyond the clinical plus CCTA model. Other unmeasured vascular and cardiometabolic factors may also contribute to the observed associations, as discussed further in the Limitations section.

An important novel aspect of this study is the observed interaction between sleep phenotype and shift-related occupation. Night-shift schedules, irregular working hours, or prolonged sedentary exposures may amplify the cardiovascular consequences of poor sleep through recurrent circadian disruption, psychosocial stress, reduced physical activity, and unfavorable dietary patterns (26). The steeper increase in predicted ischemia risk among shift-related occupations supports the concept that sleep behavior and occupational environment should be evaluated jointly when assessing cardiovascular risk in young adults.

From a clinical perspective, our findings have potential implications for early cardiovascular risk assessment. Sleep phenotype is easily obtainable and inexpensive to assess. Incorporating a simple multidimensional sleep assessment into cardiovascular risk evaluation may provide an accessible means of identifying young adults with a higher burden of subclinical coronary disease who may warrant closer risk-factor assessment and preventive attention. In particular, individuals with a poor sleep phenotype and shift-related occupations may represent a subgroup warranting closer surveillance for premature coronary disease. However, prospective studies are required before sleep phenotype can be incorporated into formal clinical decision-making.

### Limitations

Several limitations should be acknowledged. First, the retrospective observational design precludes causal inference. Reverse causality also cannot be excluded, as individuals with subclinical coronary symptoms or early ischemic discomfort may have experienced impaired sleep. Second, sleep phenotype was derived from retrospectively collected self-reported information and may be subject to recall and misclassification bias. Moreover, information regarding the duration of individual sleep characteristics, cumulative exposure to a specific sleep phenotype, and longitudinal changes in sleep patterns was not systematically available. Therefore, potential duration–response or cumulative exposure–response relationships could not be evaluated. Third, although the sleep phenotype score was prespecified using an equal-weighting approach, the relative contribution of individual sleep components was not prospectively validated. The component-level analyses performed in the present study were exploratory and therefore should be interpreted cautiously. Fourth, shift-related occupation was analyzed as a binary exposure because detailed quantitative occupational data, including night-shift frequency, hours worked per shift, duration of shift-work employment, and cumulative exposure to irregular work schedules, were not systematically available. Consequently, potential dose–response relationships between the intensity or cumulative burden of shift work and CT-derived functional ischemia could not be evaluated. Standardized quantitative information on physical activity, including exercise frequency, duration, intensity, and sedentary time, was also not systematically available and therefore could not be incorporated into the multivariable models. Consequently, residual confounding related to physical activity and sedentary behavior cannot be excluded. Socioeconomic status was also not fully available. Fifth, traditional cardiovascular risk factors, including smoking, hypertension, and diabetes, were analyzed primarily as binary variables. Detailed information regarding smoking burden, disease duration, severity, treatment intensity, and longitudinal blood pressure or glycemic control was not systematically available. Therefore, residual confounding related to cumulative risk-factor burden cannot be excluded. Furthermore, undiagnosed or asymptomatic hypertension or diabetes, particularly in this relatively young population, may have resulted in some degree of exposure misclassification. Sixth, sublingual nitroglycerin was not administered before CCTA in this cohort; therefore, the potential influence of dynamic coronary vasomotor tone or vasospasm on luminal measurements and CT-derived functional assessment cannot be completely excluded. Seventh, CT-FFR was used as a noninvasive surrogate of functional ischemia rather than invasive fractional flow reserve. Because CT-FFR is derived from CCTA anatomy and is strongly influenced by epicardial lesion characteristics, the observed association between sleep phenotype and CT-FFR cannot be interpreted as evidence of a direct functional effect of sleep disturbance independent of structural coronary disease. Formal mediation analysis was not performed; therefore, the extent to which structural plaque characteristics account for this association could not be quantified. Coronary vasospasm and microvascular dysfunction were also not directly assessed. Finally, this study involved a relatively young population from a limited geographic setting, which may affect the generalizability of the findings to other age groups, geographic regions, or ethnic populations. Moreover, because the study population consisted of young adults referred for CCTA because of suspected coronary artery disease rather than a population-based sample, individuals with cardiovascular risk factors, particularly smoking, may have been preferentially represented. Therefore, the prevalence of smoking and other risk factors in this cohort should not be extrapolated to the general young adult population. Residual confounding by unmeasured variables, such as diet, psychosocial stress, or genetic predisposition, also cannot be excluded.

## Conclusions

Poorer sleep phenotype was associated with more complex coronary plaque characteristics in unadjusted group comparisons and with a higher likelihood of CT-FFR-defined functional ischemia after adjustment for clinical confounders. These findings represent associations and should not be interpreted as evidence of a direct functional or causal effect of sleep disturbance independent of structural coronary disease. Shift-related occupation further strengthened the association between adverse sleep phenotype and CT-derived functional ischemia, highlighting the combined relevance of sleep behavior and occupational circadian disruption. Simple, inexpensive, and noninvasive assessment of sleep patterns and work schedules may have potential value in identifying younger adults with a higher burden of subclinical coronary disease, although prospective studies are required to establish causality and clinical utility.

## Additional File

The additional file for this article can be found as follows:

10.5334/gh.1586.s1 Supplementary File. Supplementary Tables 1 and 2.

## Data Availability

The raw data are available from the corresponding author upon reasonable request.
