# Supplementary material for: Sleep Phenotype, Shift-Related Occupation, and Coronary Plaque Complexity in Young Adults: Association with CT-Derived Functional Ischemia
Source: Glob Heart. 2026 Sep 22;21(1):68. doi: 10.5334/gh.1586 (PMC13614247; doi:10.5334/gh.1586)
Supplement: Supplementary file 1 [file gh-21-1-1586-s1.pdf]

Supplementary Table 1. Interobserver agreement for CCTA measurements

Measurement	Patients, n	Agreement assessment	Agreement coefficient (95% CI)	Mean bias / Overall agreement	95% limits of agreement
Degree of stenosis (%)	100	Interobserver	ICC: 0.931 (0.900–0.953)	0.8	−6.4 to 8.0
Minimum lumen area (mm^2)	100	Interobserver	ICC: 0.918 (0.881–0.944)	0.12	−0.95 to 1.19
Lesion length (mm)	100	Interobserver	ICC: 0.894 (0.848–0.927)	−0.3	−3.1 to 2.5
CACS (Agatston)	100	Interobserver	ICC: 0.956 (0.936–0.970)	1.8	−14.5 to 18.1
Diffuse lesion	100	Interobserver	Cohen's κ: 0.872 (0.775–0.969)	95.0%	—
Occlusion	100	Interobserver	Cohen's κ: 0.846 (0.637–1.000)	98.0%	—

Interobserver agreement was assessed in a randomly selected subset of 100 patients. Two experienced cardiovascular imaging readers independently reassessed the CCTA examinations while blinded to each other's measurements and to clinical characteristics, sleep phenotype, and occupational information. For continuous measurements (degree of stenosis, minimum lumen area, lesion length, and CACS), ICCs and their 95% confidence intervals were calculated using a two-way random-effects model for absolute agreement based on single measurements; mean bias and 95% limits of agreement were assessed using Bland–Altman analysis. For categorical variables (diffuse lesion and occlusion), interobserver agreement was assessed using Cohen's κ coefficients with 95% confidence intervals and overall percent agreement. CACS indicates coronary artery calcium score; CI, confidence interval; CCTA, coronary computed tomography angiography; and ICC, intraclass correlation coefficient.

Supplementary Table 2. Associations of Individual Sleep Phenotype Components With CT-FFR ≤0.80

Sleep component	Unadjusted OR (95% CI)	*P*-value	Adjusted OR (95% CI)	*P*-value	Mutually adjusted OR (95% CI)	*P*-value
Abnormal sleep duration	1.36 (1.16–1.59)	< 0.001	1.31 (1.11–1.55)	0.002	1.24 (1.04–1.48)	0.016
Late sleep timing	1.45 (1.24–1.70)	< 0.001	1.39 (1.18–1.64)	< 0.001	1.29 (1.08–1.54)	0.005
Irregular sleep pattern	1.58 (1.35–1.85)	< 0.001	1.52 (1.29–1.79)	< 0.001	1.41 (1.18–1.68)	< 0.001
Poor sleep quality / insomnia symptoms	1.33 (1.13–1.56)	< 0.001	1.27 (1.07–1.50)	0.006	1.19 (0.99–1.43)	0.061
Snoring / daytime sleepiness	1.25 (1.06–1.47)	0.007	1.21 (1.02–1.43)	0.026	1.14 (0.95–1.37)	0.153

Adjusted models included age, sex, body mass index, smoking, hypertension, diabetes, dyslipidemia, and shift-related occupation. In the mutually adjusted model, all five sleep components were entered simultaneously together with the same clinical covariates. OR indicates odds ratio; CI, confidence interval; and CT-FFR, computed tomography-derived fractional flow reserve.
